# Preparation and evaluation of *Salmonella* Enteritidis antigen conjugated with nanogold for screening of poultry flocks

**DOI:** 10.14202/vetworld.2017.848-853

**Published:** 2017-08-02

**Authors:** Hazem Mohammed Ibrahim, Rafik Hamed Sayed, Wafaa Ragab Abdel-Aziz, Rafik Tawfik Soliman

**Affiliations:** 1 Department of Bacterial Sera and Antigen Research, Veterinary Serum and Vaccine Research Institute, Abbasia, Cairo; 2 Central Laboratory for Evaluation of Veterinary Biologics, Abbasia, Cairo; 3 Department of Microbiology, Faculty of Veterinary Medicine, Cairo University, Cairo, Egypt

**Keywords:** diagnosis, lateral flow, poultry, *Salmonella* Enteritidis

## Abstract

**Aim:**

The present work aimed to develop lateral flow immunochromatographic strip (ICS) test for detection of *Salmonella* Enteritidis (SE) specific antibodies in chicken sera.

**Materials and Methods:**

A rapid lateral flow immunochromatographic test (LFIT) has been developed, in which SE Group D antigen labeled with the gold chloride molecules laid on the conjugate pad. *Staphylococcus aureus* protein A was used as capture antibody at the test line (T) of a nitrocellulose (NC) membrane and anti-SE antigen-specific rabbit antibodies were used as capture antibody at the control line (C) of the NC strip in the lateral flow layout device.

**Results:**

Using the developed LFIT, the minimal amount of SE-specific antibodies that can be detected in chicken serum sample was 1427 enzyme-linked immunosorbent assay (ELISA) unit/100 µl that was equal to 0.1 µg (Ab)/100 µl sample. 100 suspected serum samples collected from a poultry flock were tested with the prepared SE-LFIT kits and the locally prepared stained *Salmonella* antigen, and the results were compared with those obtained from examination of these samples with *Salmonella* Group D antibody ELISA kit as the gold standard test. The sensitivity, specificity, and accuracy of the prepared SE-LFIT antigen kits were 94.4%, 90%, and 94%, respectively, while those obtained with stained *Salmonella* antigen were 88.8%, 90%, and 89%, respectively.

**Conclusion:**

The developed test is a simple field rapid test of high sensitivity, specificity, and accuracy that can improve and facilitates rapid field surveillance of salmonellosis among chickens.

## Introduction

*Salmonella* spp. are among the most important agents of foodborne diseases worldwide. Human *Salmonella* outbreaks are often associated with the consumption of poultry products (meat and eggs), and one of the most prevalent serotypes associated with these products is *Salmonella* Enteritidis (SE) [1].

Poultry may carry some *Salmonella* serovars without any signs or symptoms of disease and without causing any adverse effects to the health of the bird [2]. Control of *Salmonella* infections in poultry is posing itself as one of the difficult problems not only for those who are concerned with poultry industry but also for public health hazard because most of the serovars of *Salmonella*e which poultry harbor can act as potential pathogens for man [3].

The isolation and culture of conventional detection method of *Salmonella* cannot meet the testing requirements of quick and easy detection at the grassroots level [4].

Lateral flow tests, also known as immune-chromatographic strip (ICS) tests, are point of care tests that have reduced the time spent waiting for test results from hours to minutes utilizing immunochromatographic assay. It requires no specialized equipment, less technical training for operators and has reduced the cost of device development as well as simplicity and rapidity when compared to the conventional detection methods [5]. Because of these characteristics, the ICS test is suitable for on-site testing [6]. This technique is among the most widely used technique for detection of microbial antigen in clinical specimens such as *Salmonella* Enteric serovars Typhi [7], *Vibrio cholera* [8], *Staphylococcus aureus* [9], *Yersinia* pestis in human [10], also for detection of antibodies such as anti foot-and-mouth disease (FMD) [11], antirabies [12], and anti-O FMD [13].

The present work aimed to develop lateral flow ICS test for detection of SE specific antibodies in chicken sera. The work was planned to present a simple rapid field test of high sensitivity, specificity, and accuracy that can improve and facilitates rapid field surveillance of salmonellosis among chickens.

## Materials and Methods

### Ethical approval

The approval from the Institutional Animal Ethics Committee to carry out this study was not required as no invasive technique was used.

### SE strain

SE strain was kindly obtained from Bacterial Sera and Antigen Research Department at Veterinary Serum and Vaccine Research Institute, Abbasia, Cairo, Egypt. It was used for the preparation of antigen.

### Lateral flow immunochromatographic test

#### Biochemical identification of SE bacteria strain

The biochemical identification of SE was done using analytical profile index (API) 20E plate system (Biomerieux –France cat# 20-100).

#### Preparation of the somatic (O) antigen of SE [14]

Briefly, SE culture in one slope agar was suspended in 5 ml sterile glucose broth. This suspension was added to 45 ml of sterile glucose broth in a sterile flask and thoroughly mixed and incubated at 37°C for 48 h. 2 ml of glucose broth culture was inoculated in Roux containing thiosulphate glycerin agar and incubated at 37°C for 48 h. The bacterial growth in Roux flasks was harvested using sterile buffered formal saline and sterile glass beads. The bacterial suspension was filtered by sterile gauze to remove glass beads. The filtrate was tested for purity and morphology by staining film with Gram-stain. 700 ml of absolute alcohol were added to 100 ml of bacterial suspension and left undistributed for about 36 h until precipitation was completed. The cell suspension was centrifuged for 1 h at 3000 rpm. The antigen was washed 3 times by using normal saline then adjusted to 1 mg/ml by spectrophotometry.

#### Preparation of antisera for somatic antigen prepared in rabbit [15]

Somatic (O) antigen of SE was mixed with equal volume of complete Freund’s adjuvant. The emulsion was originally injected intradermally at a dose of 0.5 mg/kg into male rabbit. Booster doses containing antigen at a dose of 0.15 mg/kg mixed with oily incomplete Freund’s adjuvant were injected S/C in the preimmunized rabbits at 2^nd^, 4^th^, 6^th^ and 8^th^ week’s intervals. After 10 days of the last injection, the serum containing rabbit polyclonal antibody specific to SE antigen.

#### Concentration and purification of polyclonal antibodies of rabbit polyclonal using the ammonium sulfate procedure [16]

The collected rabbit serum was transferred to a centrifuge tube and centrifuged for 30 min at 13,000 rpm at 4°C. The supernatant was decanted by pipetting out with a Pasteur pipette into a 25 ml beaker. The beaker (25 ml) containing the serum was placed on ice tray and placed over a magnetic stirrer. 4 ml saturated ammonium sulfate was added slowly by means of a pipette then Leaved 1 to 2 hr at 4°C to ensure precipitation of all the antibody. The solution was added to centrifuge tube and Centrifuged for 1 hr at 13,000 rpm, 4°C. Decant off the supernatant solution to beaker, while pellet was retained in the centrifuge tube. The precipitate was dissolved in a 10 ml volume of PBS and the pellet was resuspended using a glass rod. Clamp one end of the tubing using a dialysis clamp. Fill the dissolved precipitate to approximately one half of the capacity and close the tubing with a clamp. Dialysis tubing was placed in a beaker containing PBS buffer. Then Dialyzed at least 3 hour at the desired temperature (4 C) with gentle stirring of the buffer – Place the beaker on magnetic stirrer and place the stir bar in the beaker. The dialysis buffer was changed four times during dialysis and tested the access of ammonia by barium chloride 1%. The concentration of purified antibody was measured by spectrophotometer.

#### Preparation of colloidal gold nanoparticles [17]

Briefly, 1 ml of 1% (m/v) sodium citrate solution was added to 100 ml boiled deionized water. When the mixture was heated to boil again, 1 ml of 1% (m/v) gold chloride (HAuCl_4_) solution was added rapidly by constant stirring. After the color of the solution changed to wine red (in about 2 min), the solution was boiled for another 10 min. After cooling, deionized water was added until the volume reached 100 ml. The obtained gold colloid was supplemented with 0.02% (m/v) of sodium azide and stored at 4°C. The particle diameter was checked with transmission electron microscopy (H-7650).

#### Preparation of SE antigen conjugated with nanogold [18]

About 25 µl of SE antigen solution (1 mg/ml) was added into 125 µl of colloidal gold solution. The mixture was incubated for 15 min at room temperature, and then 100 µl of 10% NaCl solution was added to the previous solution. The color of samples changes from wine red to blue as the concentration of antigen decreases. The optimum concentration of antigen for colloidal gold labeling was the lowest concentration of antigen solution that did not give a change in color. The optical antigen solution was equal to 200 µl. The mixture was gently mixed for 10 min, blocked with polyethylene glycol (20,000, 1% [m/v] final concentration) by stirring for another 15 min and centrifuged at 10,000 ×*g* for 30 min. The gold pellets were suspended in 1 ml dilution buffer (20 mM Tris/HCI buffer (pH 8.2) containing 1% [w/v] bovine serum albumin, 3% [w/v] sucrose and 0.02% sodium azide) and stored at 4°C until use.

#### Preparation of lateral flow immunochromatographic test (LFIT) [18]

- Sample pad: A glass fiber was saturated with phosphate buffered saline (PBS) solution (pH 7.2) containing 0.3% Tween-20 and 0.5% (w/v) triton X100, and dried at 37°C and kept under dry conditions at room temperature until used.
- The conjugate pad: It was prepared as follows: A glass fiber was treated with 0.1% Tween-20 for 10 min and dried at 60°C. The prepared glass fiber was cut into sections (4 cm × 0.5 cm), and then saturated with 0.15 mL of SE conjugated nanogold. The conjugate pad was dried for 1 h at 37°C and stored under dry conditions at 4°C until used.
- Nitrocellulose (NC) membrane: The dispenser (BIODOT XYZ-3) was used to dispense two lines on the NC membrane (25 mm × 300 mm). The staphylococcal protein A (1.5 mg/0.1 ml) was dispensed around the bottom a the test line (1 µl/1 cm line) while rabbit antibody against SE antigen (1 mg/ml) was dispensed at the upper position as the control line (1 µl/1 cm line). The distance between two lines was 5 mm. After applying of the test line, the membrane was dried for 2-6 h in room temperature. Then, it was blocked by immersing the membrane in blocking buffer. After the whole membrane was wetted, it was washed by immersing it 5 times in the first PBS and 5 times in the second PBS solution. After that, the membrane was covered with top laminate and cut into 0.5-cm-width test-strips by using an automated cutter machine.

Two red bands at the test and control zones are developed with no further addition of reagent. If the antibodies against SE in serum sample with concentration below the detection limit, only one band at the control zone is visualized. If no band developed at both zones, the test is invalid. The intensity of the test line is in proportion to the amount of antibodies against SE present in the sample. The control zone acts as a positive control to assure that functional, conjugated antigen migrated throughout the system. The total assay time is <5 min. The estimation of the test-strip results can either be performed visually with the naked eye.

### Sensitivity of the developed LFIT

The positive standard antisera of Group D were obtained from BioChek *Salmonella* Group D antibody enzyme-linked immunosorbent assay (ELISA) (cat # CK117). The positive control was purified by using the ammonium sulfate procedure [16] to get immunoglobulins only. The purified positive control was diluted (2-fold serial dilution). The dilutions were tested by LFIT (100 µl) and *Salmonella* Group D antibody ELISA (100 µl). The ELISA procedure was carried out according to kit manual. The antibodies concentration was measured for each dilution using total protein test in spectrophotometer.

### Specificity testing of the developed LFIT

Standard antisera of *Salmonella* Pullorum (SP), *Salmonella* Typhimurium (ST), *Mycoplasma gallisepticum* (MG), *Mycoplasma synoviae* (MS), and *Escherichia coli* were tested by the prepared strip.

### Determination of specificity, sensitivity, and accuracy for LFIT and locally prepared stained SE antigen by using *Salmonella* Group D antibody

About 100 serum samples were collected from a poultry farm. The samples were tested with LFIT and locally prepared stained SE antigen also it was tested with *Salmonella* Group D antibody ELISA kit. The test ELISA procedure was done according to kit manual.

### Statistical analysis and evaluation of LFIT and locally prepared stained SE antigen (test validity)

#### Descriptive statistics

Mean and standard deviation of lateral LFIT and locally prepared stained SE antigen were determined by using the Statistix^®^ (1996) package [19].

#### Evaluation of two tests [20,21]

The evaluation of two SE tests was studied as follow (Table 1):

**Table-1:** Evaluation of diagnostic kit by using *Salmonella* Group D antibody ELISA kit (gold standard).

Test (LFIT) or local prepared antigen	*Salmonella* Group D antibody ELISA kit		

	Positive	Negative (no antibodies)	Total
+	T^+^	F^+^	T^+^F^+^
−	F^−^	T^−^	F^−^T^−^
Total	T^+^F^−^	F^+^T^−^	T^+^F^+^F^−^T^−^(n)

Gold standard: The means by which one can detect SE antibodies whether it is truly present or not. In this study, SE. antibodies detected ELISA is the gold standard. False positive (F^+^): The serum contains SE antibodies but in fact it does not contain these antibodies. False negative (F^−^): The serum does not contain SE antibodies but in fact it contains SE antibodies. True positive (T^+^): The serum contains SE antibodies and indeed contains SE antibodies. True negative (T^−^): The serum free from SE antibodies and indeed it is free. ELISA=Enzyme-linked immunosorbent assay, LFIT=Lateral flow immunochromatographic test, SE=*Salmonella* Enteritidis

- Sensitivity (true positive rate)
  Is the ability of a test to correctly identify the percentage of the serum contains SE antibodies:
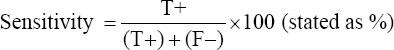
- Specificity (true negative rate)
  Is the ability of a test correctly identifies the percentage of the serum contains SE antibodies:
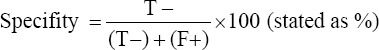
- Accuracy (validity)
  Describe the degree to which measurement reflects the true status.

## Results


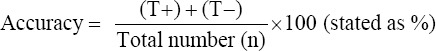


### Biochemical identification of SE bacteria strain

**Figure-1:**
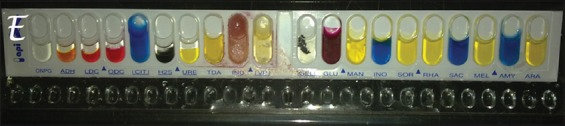
API system for biochemical identification of *Salmonella* Enteritidis bacterial strain.

The results of the biochemical identification of the *Salmonella* Enterica serotype Enteritidis (SE) strain used in the SE antigen preparation are shown in Table 2 and Figure-1.

**Table-2:** Biochemical identification of SE strain by using API system.

Biochemical test	ONPG	ADH	LDC	ODC	CIT	H2S	UREa	TDA	IND	VP	GEL	GLU	MAN	INO	SOR	RHA	SAC	MEL	AMY	ARA	OXY	NO_2_
Result	−	+	+	+	+	+	−	−	−	−	−	+	+	−	+	+	+	+	−	+	−	+

API 20E – ONPG=2-nitrophenyl-B-D-galactopyranoside, ADH=Arginine dihydrolase, LDC=Lysine decarboxylation, ODC=Ornithine decarboxylation, CIT=Citrate test, H2S=Hydrogen sulfide, UREa=Urease, TDA=Tryptophan deaminase, VP=Voges Proskauer, GEL=Gelatin liquefaction test, GLU=Glucose fermentation test, MAN=Mannitol fermentation test, INO=Inositol fermentation test, SOR=Sorbitol fermentation test, RHA=Rhamnose fermentation test, SAC=Sucrose fermentation test, MEL=Melibiose fermentation test, AMY=Amygdalin, ARA=Arabinose fermentation test, OX=Oxidase test, NO_2_=Nitrogen dioxide production, SE=*Salmonella* Enteritidis

### Sensitivity of the developed LFIT

**Figure-2:**
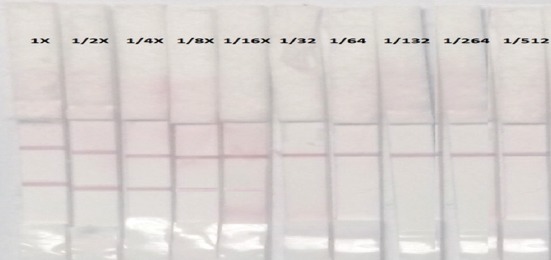
Sensitivity test of *Salmonella* Enteritidis-lateral flow immunochromatographic test.

The minimal amount of antibodies against SE in serum sample can be detected was 1427 ELISA titer/100 ml that was mean 0.1 µg (Ab)/100 ml sample as showed in Table 3 and Figure-2.

**Table-3:** The sensitivity test of SE-LFIT.

SE positive control antiserum										

2-fold serial dilution	Undiluted	1/2	1/4	1/8	1/16	1/32	1/64	1/132	1/264	1/512
ELISA titer	3779 (+ve)	3159 (+ve)	2581 (+ve)	2033 (+ve)	1427 (+ve)	917 (+ve)	514 (+ve)	124* (−ve)	33 (−ve)	0 (−ve)
SE-LFIT	+	+	+	+	+	−	−	−	−	−
Total Igs µg/100 ml	1	0.8	0.4	0.2	0.1	0.05	0.025	0.012	0.005	0.002

* According to ELISA kit interpretation: The titer under than 500 was considered negative. SE-LFIT=*Salmonella* Enteritidis-lateral flow immunochromatographic test, ELISA=Enzyme-linked immunosorbent assay, Ig=Immunoglobulin

### Specificity test of SE-LFIT

**Figure-3:**
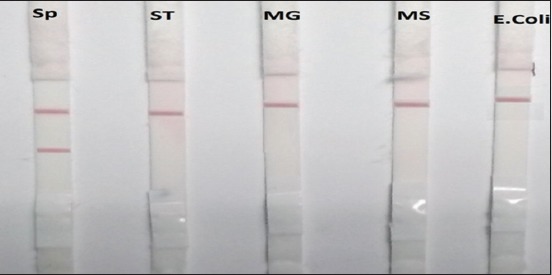
Specificity test for *Salmonella* Enteritidis-lateral flow immunochromatographic test.

The standard antisera of ST, MG, MS and *E. coli*. But SP antisera (Figure-3).

### Validity of SE-LFIT developed kits

About 100 suspected serum poultry samples were examined separately against SE antibodies using SE-LFIT, the local prepared stained antigen and *Salmonella* Group D antibody ELISA kits and the results were compared.

The results of SE-LFIT as compared with the result of bacteriological examination calculated at the (T^+^), (F^+^), (F^−^) and (T^−^) were 85, 1, 5, and 9, respectively, and for locally stained prepared antigen were 80, 1, 10, and 9, respectively, as shown in Table 4.

**Table-4:** Validity test for SE-LFIT and locally prepared stained antigen comparing with *Salmonella* Group D antibody ELISA.

Test	*Salmonella* Group D antibody ELISA			Sensitivity test (%)	Specificity test (%)	Accuracy test (%)

	+ve	−ve	Total			
SE-LFIT						
+ve	(T^+^) 85	(F^+^) 1	86	94.4	90	94
−ve	(F^-^) 5	(T^-^) 9	14			
Total	90	10	100			
Locally prepared stained antigen						
+ve	(T^+^) 80	(F^+^) 1	81	88.8	90	89
−ve	(F^−^) 10	(T^−^) 9	19			
Total	90	10	100			

SE-LFIT=*Salmonella* Enteritidis-lateral flow immunochromatographic test

The sensitivity, specificity and accuracy of SE-LFIT as compared to ELISA were calculated and was found to be 94.4%, 90% and 94%, respectively, and for locally prepared stained antigen 88.8%, 90% and 89%, respectively, as shown in Table 4.

## Discussion

*Salmonella* infections are considered to be the most important disease affecting poultry. The disease causes severe damage among young birds with a high mortality rate. Adult birds are often chronic carriers of *Salmonella* organisms without outward signs. In this work, specific Group D antigen was prepared and conjugated with nanogold that was laid on lateral flow ICS (LFIT).

The ideal diagnostic tool of SE should be able to detect the antibody in the shortest possible time, simple, sensitive, specific and inexpensive. Furthermore, it should be suitable as field test or laboratory test and can be applied on large scale of poultry.

The LFIT was low cost, fast, no requirements for skilled technicians and applicable in field condition gives results within 5 min that is helpful in large poultry flock *Salmonella* screening. Unfortunately, the LFIT did not have ability to differentiate between SE and SP, as there is cross-reactivity between them as the both SE and SP are sharing the same somatic antigen.

The minimal amount of antibodies against SE in poultry serum can be detected using SE-LFIT was 1427 ELISA titer/100 ml or 0.1 µg/100 µl in samples. Biagini *et al*. [22] detected 3 mg/100 ml anti-anthrax immunoglobulin G in bovine serum.

The developed SE-LFIT and locally prepared antigen were validated by testing 100 poultry serum samples in comparison with *Salmonella* Group D antibody ELISA kit. The sensitivity, specificity and accuracy of SE-LFIT as compared to ELISA were 94.4%, 90%, and 94%, respectively, but were 88.8% 90% and 89%, respectively, for locally prepared stained antigen that was lower than SE-LFIT. The prepared SE-LFIT is to be used for detection of SE antibody in poultry serum as poultry serum also contains antibody against MG, MS and *E. coli*, so we have to check if there is cross reaction between them or not in poultry serum regardless of affected organ.

These results are similar to those recorded by Yang *et al*. [13] who found that the sensitivity and specificity of lateral flow test for detection of anti-FMD virus compared to anti-FMD ELISA kit were 100% and 99.1%, respectively. A sensitivity rate of 100% and a specificity of 98.8% of the lateral flow strips developed for detection of anti-O serotype FMD compared to ELISA kit were reported [11]. Furthermore, there was a lateral flow test sensitivity and specificity of 88.7% and 91.9%, respectively, for the detection of anti-rabies as compared with ELISA [12].

Compared with the commercially available SE antibodies diagnostic tools. The developed SE-LFIT is not only very rapid test (5 min) but also it is simple, convenient, has long shelf time and can be used by untrained personal at poultry farm site without requirement of additional equipment. Moreover, the SE-LFIT preparation technology has been strongly improved, which will be reflected on its sensitivity and specificity. These tools are badly required for routine diagnosis in the laboratory and under field conditions.

## Conclusion

The developed test is a simple field rapid test of high sensitivity, specificity, and accuracy that can improve and facilitates rapid field surveillance of salmonellosis among chickens.

## Authors’ Contributions

HMI and RHS designed the work. HMI, RHS, and WRAE conducted the research work. Data analysis and manuscript were written by HMI, RHS and WRA under the guidance of RTS. All authors read and approved the final manuscript.

## References

[1] Borges K.A, Furian T.Q, de Souza S.N, Tondo E.C, Streck A.F, Salle C.T, de Souza Moraes H.L, do Nascimento V.P (2017). Spread of a major clone of *Salmonella* Enterica serotype enteritidis in poultry and in Salmonellosis outbreaks in southern Brazil. J. Food Protect.

[2] Jarquin R, Hanning I, Ahn S, Ricke S.C (2009). Development of rapid detection and genetic characterization of *Salmonella* in poultry breeder feeds. Sens. (Basel).

[3] Galis A.M, Marcq C, Marlier D, Portetelle D, Van I, Beckers Y, Thewis A (2013). Control of *Salmonella* contamination of shell eggs-preharvest and postharvest methods: A review. Compr. Rev. Food Sci. Food Saf.

[4] Wu B, Zhang X, Pan W, Zhang L, Zhang F (2015). Determination of *Salmonella* Pullorum with nanoparticles immune based lateral flow strip assay. Adv. Microbiol.

[5] Chen W, Rahman R.T, Antora R.A, Saqib N, Hossain P, Zhang J, Al-Hajj N.Q, Lou Z, Zaixiang L (2015). Lateral flow test strip approaches for rapid detection of food contaminants and pathogens: Review. Am. Res. Thoughts.

[6] Lata K, Naik L, Sharma R, Rajput Y.S (2013). Lateral flow assay-concept and its applications in food analysis. Indian Food Ind. Mag.

[7] Pandey S.K, Suri C.R, Chaudhry M, Tiwari R.P, Praveen R (2012). A gold nanoparticles based immuno-bioprobe for detection of Vi capsular polysaccharide of *Salmonella* Enterica *Serovars typhi*. Mol. Biosys.

[8] Shyu R, Tang S, Chiao D, Hung Y (2010). Gold nanoparticle-based lateral flow assay for detection of staphylococcal enterotoxin B. Food Chem.

[9] Hong W, Lihua H, Haoran W, Jianfeng Q, Zhaobiao G, Chengke X, Ziwen Z, Youbao Z, Zongmin D, Yanfeng Y, Yan Z, Huijie H, Ruifu Y, Lei Z (2010). Development of an up converting phosphor technology-based 10-channel lateral flow assay for profiling antibodies against *Yersinia pestis*. J. Microbiol. Methods.

[10] Chen W, Zhang J, Lu G, Yuan Z, Wu Q, Li J (2014). Development of an immunochromatographic lateral flow device for rapid diagnosis of *Vibrio cholerae* O1 serotype Ogawa. Clin. Biochem.

[11] Oem J.K, Ferris N.P, Kwang-Nyeong L, Yi-Seok J, Bang-Hun H, Jong-Hyeon P (2009). Simple and rapid lateral-flow assay for the detection of foot-and-mouth disease virus. Clin. Vaccine Immunol.

[12] Shiota S, Mannen K, Matsumoto T (2009). Development and evaluation of a rapid neutralizing antibody test for rabies. J. Virol. Methods.

[13] Yang W, Li X, Liu G, Zhang B, Zhang Y, Kong T, Tang J, Li D, Wang Z (2011). A colloidal gold prob-based silver enhancement immune-chromatographic assay for the rapid detection of abrin-a. Biosens. Bioelectron.

[14] Wary C, Wary A (2000). *Salmonella* in domestic animals.

[15] Gulbenkian S, Wharton J, Polak J.M (1987). The visualization of cardiovascular innervations in the guinea-pig using an antiserum to protein gene product 9.5. J. Auton. Nerv. Syst.

[16] Donovan J, Brown P (1995). Current Protocols in Immunology. Wiley Online Library.

[17] Singh J, Sharma S, Seema N (2015). Evaluation of gold nanoparticle based lateral flow assays for diagnosis of *Enterobacteriaceae* members in food and water. Food Chem.

[18] Singh J, Sharma S, Seema N (2015). Nanogold based lateral flow assay for the detection of *Salmonella* Typhi in environmental water samples. Anal Methods.

[19] (1996). Statistix.

[20] Timmreck T.C (1994). An Introduction to Epidemiology.

[21] Thrusfield M (2007). Veterinary Epidemiology.

[22] Biagini R.E, Sammons D.L, Smith J.P, MacKenzie B.A, Striley C.A.F, Snawder J.E, Robertson S.A, Quinn C.P (2006). Rapid, sensitive and specific lateral flow immunochromatographic device to measure anti-anthrax protective antigen immunoglobulin in serum and whole blood. Clin Vaccine Immunol.

